# Incidence, Risk Factors and Outcomes of Postoperative Headache After Stanford Type a Acute Aortic Dissection Surgery

**DOI:** 10.3389/fcvm.2021.781137

**Published:** 2021-12-23

**Authors:** Dashuai Wang, Sheng Le, Jingjing Luo, Xing Chen, Rui Li, Jia Wu, Yu Song, Fei Xie, Ximei Li, Hongfei Wang, Xiaofan Huang, Ping Ye, Xinling Du, Anchen Zhang

**Affiliations:** ^1^Department of Cardiovascular Surgery, Union Hospital, Tongji Medical College, Huazhong University of Science and Technology, Wuhan, China; ^2^Key Laboratory for Molecular Diagnosis of Hubei Province, The Central Hospital of Wuhan, Tongji Medical College, Huazhong University of Science and Technology, Wuhan, China; ^3^Department of Cardiovascular Surgery, First Affiliated Hospital of Zhengzhou University, Zhengzhou, China; ^4^Department of Nursing, Huaihe Hospital of Henan University, Kaifeng, China; ^5^Department of Cardiology, The Central Hospital of Wuhan, Tongji Medical College, Huazhong University of Science and Technology, Wuhan, China

**Keywords:** headache, cardiac surgery, Stanford type A acute aortic dissection, risk factor, nomogram

## Abstract

**Background:** Postoperative headache (POH) is common in clinical practice, however, no studies about POH after Stanford type A acute aortic dissection surgery (AADS) exist. This study aims to describe the incidence, risk factors and outcomes of POH after AADS, and to construct two prediction models.

**Methods:** Adults who underwent AADS from 2016 to 2020 in four tertiary hospitals were enrolled. Training and validation sets were randomly assigned according to a 7:3 ratio. Risk factors were identified by univariate and multivariate logistic regression analysis. Nomograms were constructed and validated on the basis of independent predictors.

**Results:** POH developed in 380 of the 1,476 included patients (25.7%). Poorer outcomes were observed in patients with POH. Eight independent predictors for POH after AADS were identified when both preoperative and intraoperative variables were analyzed, including younger age, female sex, smoking history, chronic headache history, cerebrovascular disease, use of deep hypothermic circulatory arrest, more blood transfusion, and longer cardiopulmonary bypass time. White blood cell and platelet count were also identified as significant predictors when intraoperative variables were excluded from the multivariate analysis. A full nomogram and a preoperative nomogram were constructed based on these independent predictors, both demonstrating good discrimination, calibration, clinical usefulness, and were well validated. Risk stratification was performed and three risk intervals were defined based on the full nomogram and clinical practice.

**Conclusions:** POH was common after AADS, portending poorer outcomes. Two nomograms predicting POH were developed and validated, which may have clinical utility in risk evaluation, early prevention, and doctor-patient communication.

## Introduction

Stanford type A acute aortic dissection is a life-threatening cardiovascular disease related to significant risk of morbidity and mortality (1). Although great improvement has been made in diagnostic techniques and initial management over the past decades, prompt surgical interventions remain the standard treatment (2). However, survival after Stanford type A acute aortic dissection surgery (AADS) is still suboptimal and a considerable proportion of patients develop various postoperative complications (1).

Postoperative headache (POH) is one of the most common surgical complications, which is associated with decreased quality of life, poorer outcomes, and additional economic burden (3–7). At present, many studies describing the incidence and outcomes of POH have been conducted and several independent risk factors for POH have been reported in the literature (7–12). Nevertheless, none of these studies were carried out in patients undergoing AADS and available information is still lacking in this population.

In the present study, we aimed to investigate the incidence, risk factors and outcomes of POH in adult patients who underwent AADS, and to construct and validate two nomogram models for POH after AADS to provide help for risk assessment and early prevention.

## Materials and Methods

### Ethical Statement

This study was conducted according to the ethical statement of the Declaration of Helsinki. The Ethics Committee of Tongji Medical College of Huazhong University of Science and Technology (IORG No. IORG0003571) approved this study. Written informed consent was waived due to its observational, retrospective nature.

### Study Population

This was a multicenter, observational, retrospective study. Consecutive adult patients (older than 18 years) who underwent AADS in four tertiary care centers between 2016 and 2020 were enrolled. Patients with the following conditions were excluded from the study: (1) intraoperative death or postoperative unconsciousness, and (2) records with missing data.

### Data Collection and Variables

We collected clinical data using the hospital's electronic medical record management systems. Pre-, intra-, and post-operative variables were collected and analyzed. Preoperative variables included sex, age, body mass index, smoking, drinking, diabetes mellitus, hypertension, cerebrovascular disease, chronic headache history, chronic obstructive pulmonary disease, gastrointestinal tract disease, peripheral vascular disease, atrial fibrillation, left ventricular ejection fraction, cardiac function, pulmonary artery hypertension, pericardial effusion, general surgery history, cardiac surgery history, red blood cell (RBC) count, white blood cell (WBC) count, platelet count, hemoglobin, serum creatinine, urea nitrogen, uric acid, albumin, and globulin levels. Intraoperative variables included cardiopulmonary bypass (CPB) time, aortic cross clamp time, use of deep hypothermic circulatory arrest (DHCA), and transfusion of RBC. Postoperative variables included readmission to intensive care unit (ICU), reintubation, tracheotomy, mortality, ICU duration, and hospital stay.

### Definitions of Important Variables

In this study, POH was diagnosed on the basis of a self-reported or recorded headache identified in the electronic medical records. Body mass index was calculated on the basis of height and body weight. Smoking history referred to current or previous daily smoking. Chronic obstructive pulmonary disease was defined in accordance with previous diagnosis, self-report, or FEV1/FVC ≤ 0.7. Chronic headache history referred to recorded or self-reported migraine or other kinds of recurrent headaches. Cerebrovascular disease referred to a history of carotid artery surgery, transient ischemic attack, cerebral infarction, cerebral hemorrhage or stroke. Diabetes mellitus referred to fasting glucose ≥ 7.0 mmol/L, random glucose ≥ 11.1 mmol/L, or previous diagnosis of diabetes mellitus. Hypertension referred to previous diagnosis of hypertension or blood pressure > 140/90 mmHg.

### Statistical Analysis

Patients were divided into the training set and the validation set by 7:3 ratio. The development and internal validation of the model was performed using the training set and the external validation of the model was performed using the independent validation set. Normally distributed continuous variables were presented as means with standard deviations. Non-normally distributed continuous variables were presented as medians with inter-quartile ranges. Categorical variables were presented as frequencies with percentages. We first performed univariate logistic regression analysis to screen possible risk factors. Factors with *P* < 0.1 or considered to be clinically significant were further entered into a forward stepwise multivariate logistic regression analysis to identify independent risk factors. A nomogram based on these independent risk factors was then constructed.

We performed internal validation by bootstrap method using 1,000 replicates in the training set and external validation in the validation set. The area under the receiver operating characteristic (ROC) curve (AUC) was used to assess the discrimination. Both Hosmer-Lemeshow goodness-of-fit test and visual inspection were used to assess the calibration. Decision curves and clinical impact curves were used to assess the clinical usefulness. The Delong method was used to compare the AUCs between the training and the validation sets (13).

Statistical analyses were performed using SPSS (IBM SPSS Statistics 26.0, SPSS Inc., Chicago, IL) and R software (version 4.0.5, www.R-project.org/). A two-tailed *P*-value <0.05 was deemed statistically significant.

## Results

### Demographic Characteristics

**Figure 1:**
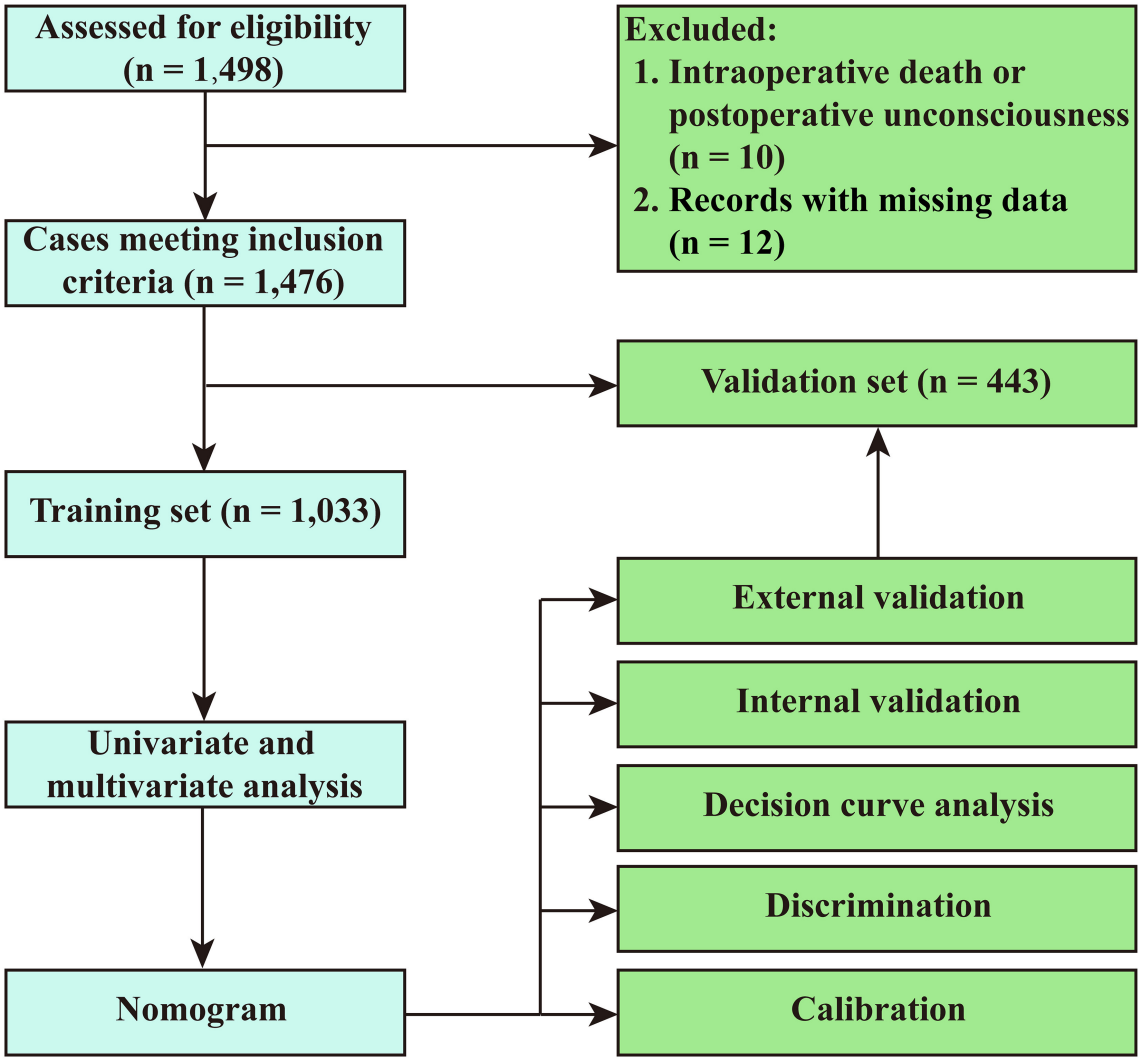
Flow chart of the study.

Among the 1,498 adults who underwent AADS, 10 patients died intraoperatively or lapsed into unconsciousness postoperatively, and 12 patients had missing data in the medical records (Figure 1). The remaining 1,476 patients meeting the inclusion criteria were divided into two groups based on if one or two episodes of headache developed during their postoperative hospitalization and were further analyzed. The mean age of these included patients was 50.83 ± 11.35 years, 75.6% were men. The overall morbidity of POH after AADS was 25.7%.

There were multiple comorbidities and underlying conditions in this study population, in which smoking history existed in 43.9% of the patients, drinking history in 35.8%, hypertension in 68.1%, diabetes mellitus in 4.3%, cerebrovascular disease in 17.8%, chronic headache history in 11.4%, peripheral vascular disease in 13.6%, gastrointestinal tract disease in 8.5%, chronic obstructive pulmonary disease in 1.0%, atrial fibrillation in 0.8%, general surgery history in 20.5%, cardiac surgery history in 6.5%, pulmonary artery hypertension in 2.8%, pericardial effusion in 27.0%. The median CPB time was 204 (166, 247) min, aortic cross clamp time was 116 (94, 143) min, intraoperative RBC transfusion was 4.0 (2.5, 6.0) units, and DHCA was used in 58.9% of the patients. No significant difference was observed with regard to baseline conditions and operative variables between the training set and the validation set (Table 1).

**Table 1:** Comparison of characteristics between the training and validation sets.

**Characteristics**	**Training set**	**Validation set**	***P* value**
	***n* = 1,033 (%)**	***n* = 443 (%)**	
**Demographics**			
Male	787 (76.2)	329 (74.3)	0.431
Age (years)	50.62 ± 11.56	51.29 ± 10.85	0.299
Body mass index (kg/m^2^)	25.29 ± 3.65	25.62 ± 3.62	0.112
Smoking history	470 (45.5)	178 (40.2)	0.059
Drinking history	367 (35.5)	161 (36.3)	0.764
**Underlying conditions**			
Hypertension	696 (67.4)	309 (69.8)	0.370
Diabetes mellitus	42 (4.1)	21 (4.7)	0.557
Chronic obstructive pulmonary disease	13 (1.3)	2 (0.5)	0.157
Cerebrovascular disease	184 (17.8)	79 (17.8)	0.992
Peripheral vascular disease	137 (13.3)	64 (14.4)	0.543
Gastrointestinal tract disease	88 (8.5)	38 (8.6)	0.970
Atrial fibrillation	10 (1.0)	2 (0.5)	0.311
Cardiac surgery history	70 (6.8)	26 (5.9)	0.517
General surgery history	207 (20.0)	96 (21.7)	0.477
Pulmonary artery hypertension	28 (2.7)	14 (3.2)	0.634
Pericardial effusion	271 (26.2)	128 (28.9)	0.292
NYHA class III-IV	87 (8.4)	36 (8.1)	0.851
Left ventricular ejection fraction (%)	62 (59, 65)	61 (59, 65)	0.460
**Laboratory values**			
White blood cell count (×10^9^/L)	10.09 (7.57, 12.89)	10.24 (7.43, 12.85)	0.802
Red blood cell count (×10^12^/L)	4.28 (3.85, 4.63)	4.25 (3.81, 4.62)	0.930
Hemoglobin (g/l)	129 (116, 140)	130 (116, 140)	0.881
Platelet count (×10^9^/L)	160 (128, 206)	159 (128, 205)	0.480
Serum creatinine (μmol/L)	80.7 (65.5, 110.8)	81.5 (66.8, 114)	0.437
Urea nitrogen (mmol/L)	6.20 (4.99, 7.91)	6.31 (5.20, 8.00)	0.149
Uric acid (μmol/L)	369.2 (287.2, 456.5)	366.9 (279.8, 467.8)	0.936
Serum albumin (g/L)	37.9 (35.0, 40.9)	37.7 (34.7, 40.7)	0.284
Serum globulin (g/L)	25.3 (22.6, 28.3)	26.0 (22.9, 28.3)	0.295
**Operative variables**			
Cardiopulmonary bypass time (minutes)	205 (167, 248)	202 (165, 246)	0.527
Aortic cross clamp time (minutes)	116 (95, 145)	116 (91, 141)	0.168
Deep hypothermic circulatory arrest	611 (59.1)	259 (58.5)	0.807
Transfusion of red blood cells (units)	4 (2, 6)	4 (3, 6)	0.272

### Development of the Full Nomogram

**Figure 2:**
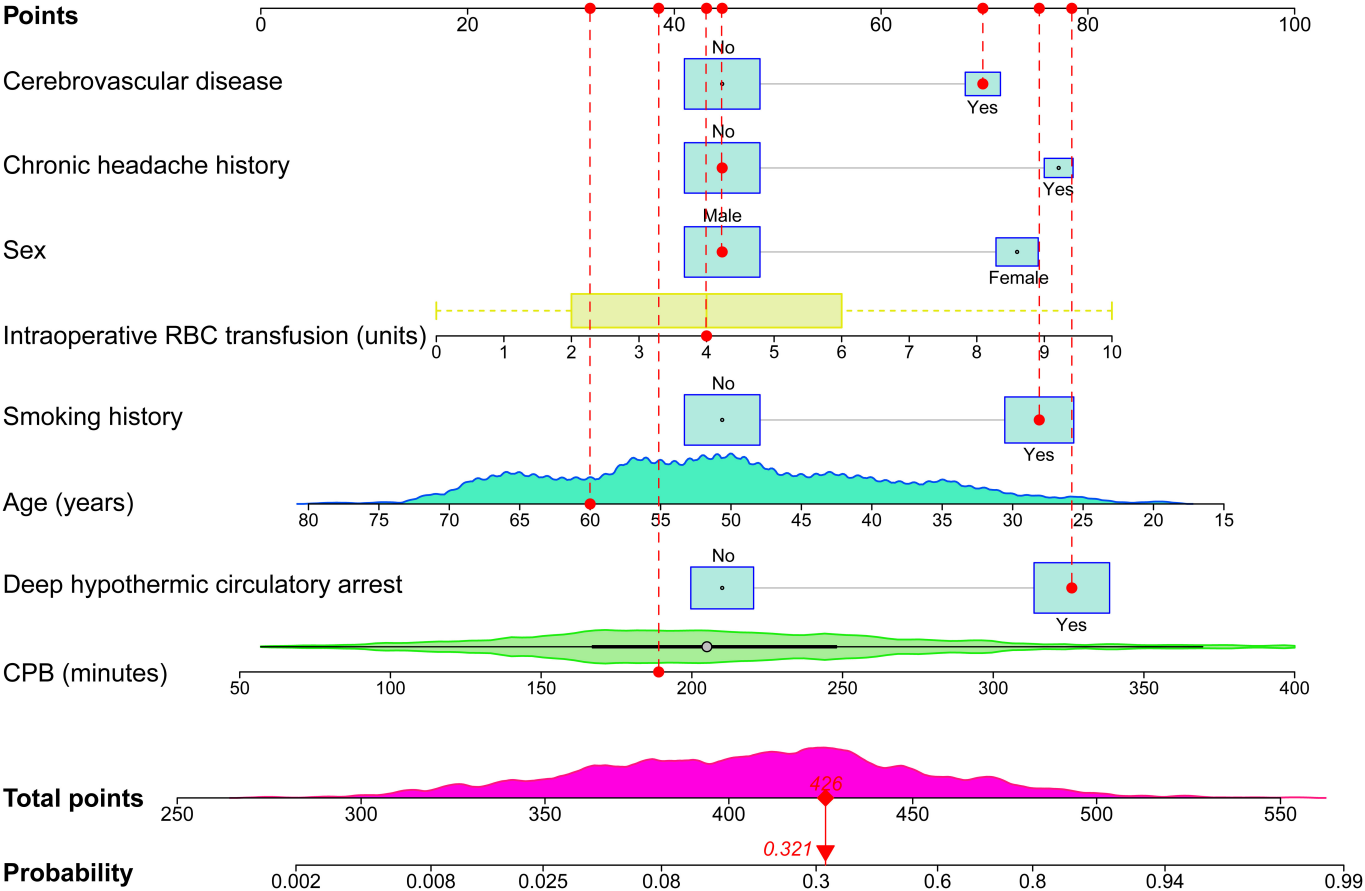
Full nomogram for the prediction of POH after AADS. A concrete case is presented to show how to use the nomogram. This was a 60-year-old male patient who had a history of smoking and cerebrovascular disease, but did not have a chronic headache history. He experienced DHCA, and the duration of CPB was 189 min and the transfused RBC was 4 units. The individual item score corresponding to each factor was presented at the top, and the total points were obtained from the sum of the scores corresponding to each factor by a red dot. Given values of the 8 predictors, the patient can be intuitively mapped onto the nomogram. It can be clearly seen from the nomogram that the total points of this patient was 426 points and the corresponding probability of POH was 0.321. AADS, Stanford type A acute aortic dissection surgery; CPB, cardiopulmonary bypass; DHCA, deep hypothermic circulatory arrest; POH, postoperative headache; RBC, red blood cell.

The results of the univariate analysis conducted in the training set are presented in Table 2. Factors with *P* < 0.1 or considered to be clinically significant were further entered into a forward stepwise multivariate logistic regression analysis to identify independent risk factors. Co-linearity of covariates were assessed and highly collinear covariates were removed from the model. Finally, eight independent risk factors were identified in the full model, including younger age, female sex, smoking history, cerebrovascular disease, chronic headache history, the use of DHCA, CPB time, and intraoperative RBC transfusion (Table 3). A full nomogram used to predict the probability of POH after AADS was then constructed based on these preoperative and intraoperative predictors (Figure 2). The nomogram scaled each regression coefficient to a scale of 0–100 points, which demonstrated their relative importance. We also created an interactive web-based dynamic nomogram which is available online (https://xinlingdu.shinyapps.io/dynnomapp/).

**Table 2:** Univariate analysis of possible risk factors for POH after AADS in the training set.

**Characteristics**	**Without POH**	**With POH**	***P* value**
	***n* = 756 (%)**	***n* = 277 (%)**	
**Demographics**			
Male	581 (76.9)	206 (74.4)	0.406
Age (years)	51.92 ± 11.33	47.09 ± 11.46	<0.001
Body mass index (kg/m^2^)	25.34 ± 3.58	25.13 ± 3.84	0.409
Smoking history	309 (40.9)	161 (58.1)	<0.001
Drinking history	254 (33.6)	113 (40.8)	0.032
**Underlying conditions**			
Hypertension	534 (70.6)	162 (58.5)	<0.001
Diabetes mellitus	29 (3.8)	13 (4.7)	0.537
Chronic headache history	68 (9.0)	63 (22.7)	<0.001
Chronic obstructive pulmonary disease	12 (1.6)	1 (0.4)	0.117
Cerebrovascular disease	115 (15.2)	69 (24.9)	<0.001
Peripheral vascular disease	103 (13.6)	34 (12.3)	0.571
Gastrointestinal tract disease	63 (8.3)	25 (9.0)	0.724
Atrial fibrillation	9 (1.2)	1 (0.4)	0.228
Cardiac surgery history	42 (5.6)	28 (10.1)	0.010
General surgery history	156 (20.6)	51 (18.4)	0.429
Pulmonary artery hypertension	21 (2.8)	7 (2.5)	0.826
Pericardial effusion	191 (25.3)	80 (28.9)	0.242
NYHA class III-IV	65 (8.6)	22 (7.9)	0.737
Left ventricular ejection fraction (%)	62 (59, 65)	61 (59, 65)	0.336
**Laboratory values**			
White blood cell count (×10^9^/L)	9.97 (7.42, 12.37)	10.45 (7.92, 14.24)	0.002
Red blood cell count (×10^12^/L)	4.29 (3.86, 4.63)	4.22 (3.77, 4.62)	0.506
Hemoglobin (g/l)	130 (116, 140)	128 (114, 141)	0.272
Platelet count (×10^9^/L)	165 (130, 211)	153 (125, 194)	0.007
Serum creatinine (μmol/L)	80.9 (66.7, 108.3)	79.5 (63.7, 121.4)	0.880
Urea nitrogen (mmol/L)	6.26 (5.04, 7.80)	6.20 (4.88, 8.65)	0.860
Uric acid (μmol/L)	365.6 (287.6, 444.4)	375.0 (283.3, 496.6)	0.275
Serum albumin (g/L)	38.1 (35.0, 41.0)	37.4 (34.4, 40.2)	0.017
Serum globulin (g/L)	25.4 (22.9, 28.3)	25.0 (22.5, 28.2)	0.350
**Operative variables**			
Cardiopulmonary bypass time (minutes)	193 (159, 234)	243 (198, 288)	<0.001
Aortic cross clamp time (minutes)	112 (92, 137)	138 (111, 168)	<0.001
Deep hypothermic circulatory arrest	392 (51.9)	219 (79.1)	0.807
Transfusion of red blood cells (units)	4 (2, 5)	6 (3, 7)	<0.001

*AADS, Stanford type A acute aortic dissection surgery; POH, postoperative headache*.

**Table 3:** Multivariate analysis of independent risk factors for POH after AADS.

**Characteristics**	**Coefficient**	**Standard error**	**OR (95% CI)**	***P* value**
Female sex	1.082	0.247	2.951 (1.817–4.792)	<0.001
Age (years)	−0.052	0.008	0.950 (0.935–0.965)	<0.001
Smoking history	1.163	0.210	3.201 (2.121–4.831)	<0.001
Chronic headache history	1.235	0.239	3.437 (2.150–5.495)	<0.001
Cerebrovascular disease	0.956	0.222	2.602 (1.685–4.019)	<0.001
Cardiopulmonary bypass time (min)	0.011	0.002	1.011 (1.008–1.014)	<0.001
Intraoperative transfusion of RBC (units)	0.248	0.042	1.281 (1.181–1.390)	<0.001
Deep hypothermic circulatory arrest	1.283	0.195	3.607 (2.459–5.290)	<0.001
Constant	−4.134	0.559	0.016	<0.001

*AADS, Stanford type A acute aortic dissection surgery; CI, confidence interval; OR, odds ratio; POH, postoperative headache; RBC, red blood cell*.

The probability range of POH after AADS predicted by the nomogram was large. The personalized risk can be directly and easily assessed by summing the points of all the predictors. Young females who had a history of smoking, cerebrovascular disease, chronic headache, longer CPB time, more intraoperative RBC transfusion, and experienced DHCA may obtain more points and thus at a higher risk of POH. A concrete case is illustrated in Figure 2. For the online predictive system, press the “Quit” button in the bottom-left corner to exit the application and reload the procedure. Fill in the information of a concrete patients and click the “Predict” button, the predicted probability of POH after AADS was presented in the “Graphical summary” area on the right. The information of the patient and the model can also be acquired by clicking the “Numerical summary” and “Model summary” (Supplementary Figure 1).

### Validation and Assessment of the Full Nomogram

**Figure 3:**
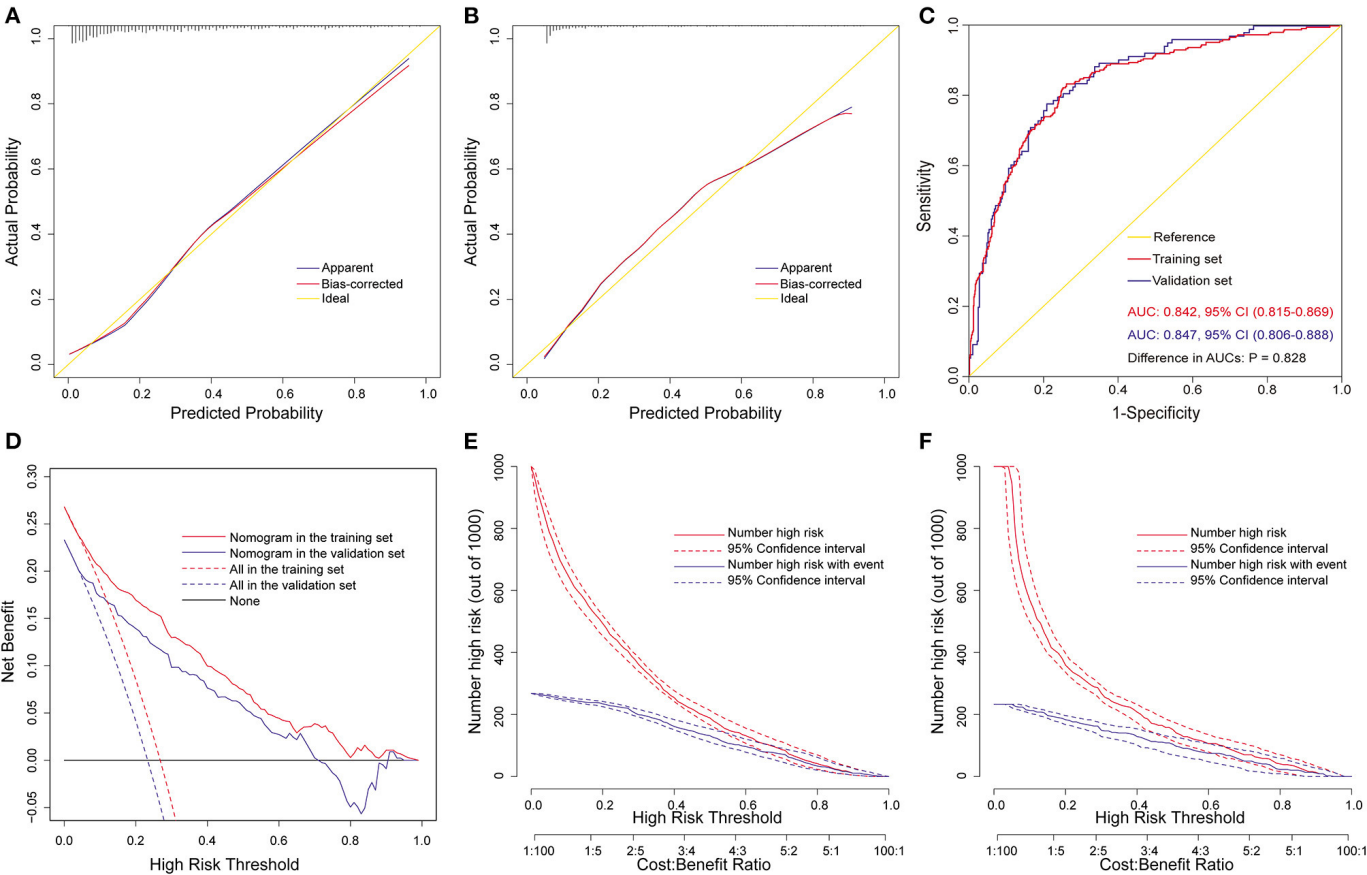
Assessment of the full nomogram model for POH after AADS. Calibration plots in the training set **(A)** and the validation set **(B)**, ROC curves in the two sets **(C)**, decision curves in the two sets **(D)**, and clinical impact curves in the training set **(E)** and the validation set **(F)**. AADS, Stanford type A acute aortic dissection surgery; AUC, area under the receiver operating characteristic curve; CI, confidence interval; POH, postoperative headache; ROC, receiver operating characteristic curve.

The nomogram was well validated by both internal and external validations. By visual inspection, the calibration curves showed good consistency between estimated and actual probabilities. This was in agreement with the results of the goodness-of-fit test, with Hosmer-Lemeshow chi-square statistics of 5.226 (*P* = 0.733, Figure 3A) and 3.134 (*P* = 0.926, Figure 3B) in the training and validation sets. ROC curves were plotted to assess the discrimination, and the AUCs were 0.842 [95% confidence interval (CI), 0.815–0.869] and 0.847 (95% CI, 0.806–0.888) in the training and validation sets (Figure 3C), both indicating excellent predictive capability. There was no significant difference between the two AUCs (*P* = 0.83). Decision curve analysis was carried out to evaluate the clinical usefulness of the nomogram. The decision curves indicated that the nomogram could obtain more net benefits across a wide range of threshold probabilities than either the treat-none scheme or the treat-all-patients scheme both in the training and validation sets (Figure 3D). The clinical impact curves also revealed that the nomogram was clinically useful (Figures 3E,F).

### Development, Validation, and Assessment of the Preoperative Nomogram

**Figure 4:**
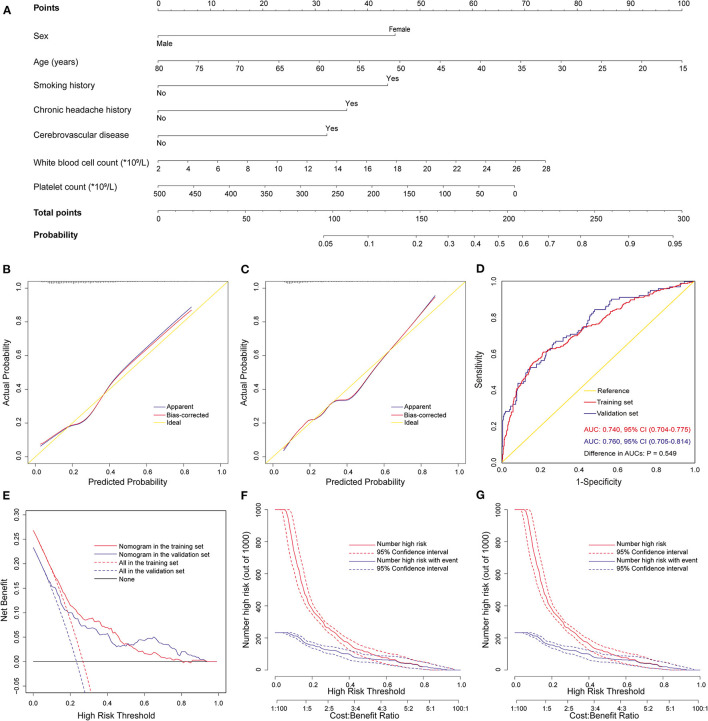
Development, validation, and assessment of the preoperative nomogram for POH after AADS. The construction of the preoperative nomogram for POH after AADS **(A)**, calibration plots in the training set **(B)** and the validation set **(C)**, ROC curves in the two sets **(D)**, decision curves in the two sets **(E)**, and clinical impact curves in the training set **(F)** and the validation set **(G)**. AADS, Stanford type A acute aortic dissection surgery; AUC, area under the receiver operating characteristic curve; CI, confidence interval; POH, postoperative headache; ROC, receiver operating characteristic curve.

The above nomogram model was established using both preoperative and intraoperative variables. To facilitate early preoperative prediction, we further established a preoperative nomogram using only preoperative factors. Seven independent predictors were identified by multivariate logistic regression analysis in the training set, including lower platelet count, higher WBC count, and the five preoperative predictors mentioned above (Table 4). Then, a preoperative nomogram was constructed on the basis of these predictors (Figure 4A).

**Table 4:** Multivariate analysis of preoperative independent risk factors for POH after AADS.

**Characteristics**	**Coefficient**	**Standard**	**OR (95% CI)**	***P* value**
		**error**		
Platelet count (×10^9^/L)	−0.004	0.001	0.996 (0.994–0.998)	0.002
Female sex	1.332	0.228	3.789 (2.423–5.927)	<0.001
Age (years)	−0.045	0.007	0.956 (0.942–0.969)	<0.001
Smoking history	1.291	0.194	3.636 (2.488–5.313)	<0.001
Cerebrovascular disease	0.949	0.198	2.584 (1.753–3.808)	<0.001
Chronic headache history	1.060	0.217	2.888 (1.888–4.418)	<0.001
White blood cell count (×10^9^/L)	0.084	0.020	1.087 (1.045–1.132)	<0.001
Constant	−1.610	0.625	0.200	0.010

*AADS, Stanford type A acute aortic dissection surgery; CI, confidence interval; OR, odds ratio; POH, postoperative headache*.

This nomogram was also well validated by both internal validation using bootstrap method in the training set and external validation in the independent validation set. The model fitted well by visual inspection of the calibration curves and goodness-of-fit test, with Hosmer-Lemeshow chi-square statistics of 7.998 (*P* = 0.434, Figure 4B) and 10.148 (*P* = 0.255, Figure 4C) in the training and validation sets, respectively. The AUCs were respectively 0.740 (95% CI, 0.704–0.775) and 0.760 (95% CI, 0.705–0.814) in the training and validation sets, indicating no significant difference (*P* = 0.55, Figure 4D). The decision and clinical impact curves also demonstrated that the nomogram may have usefulness in clinical practice.

### Risk Stratification

**Figure 5:**
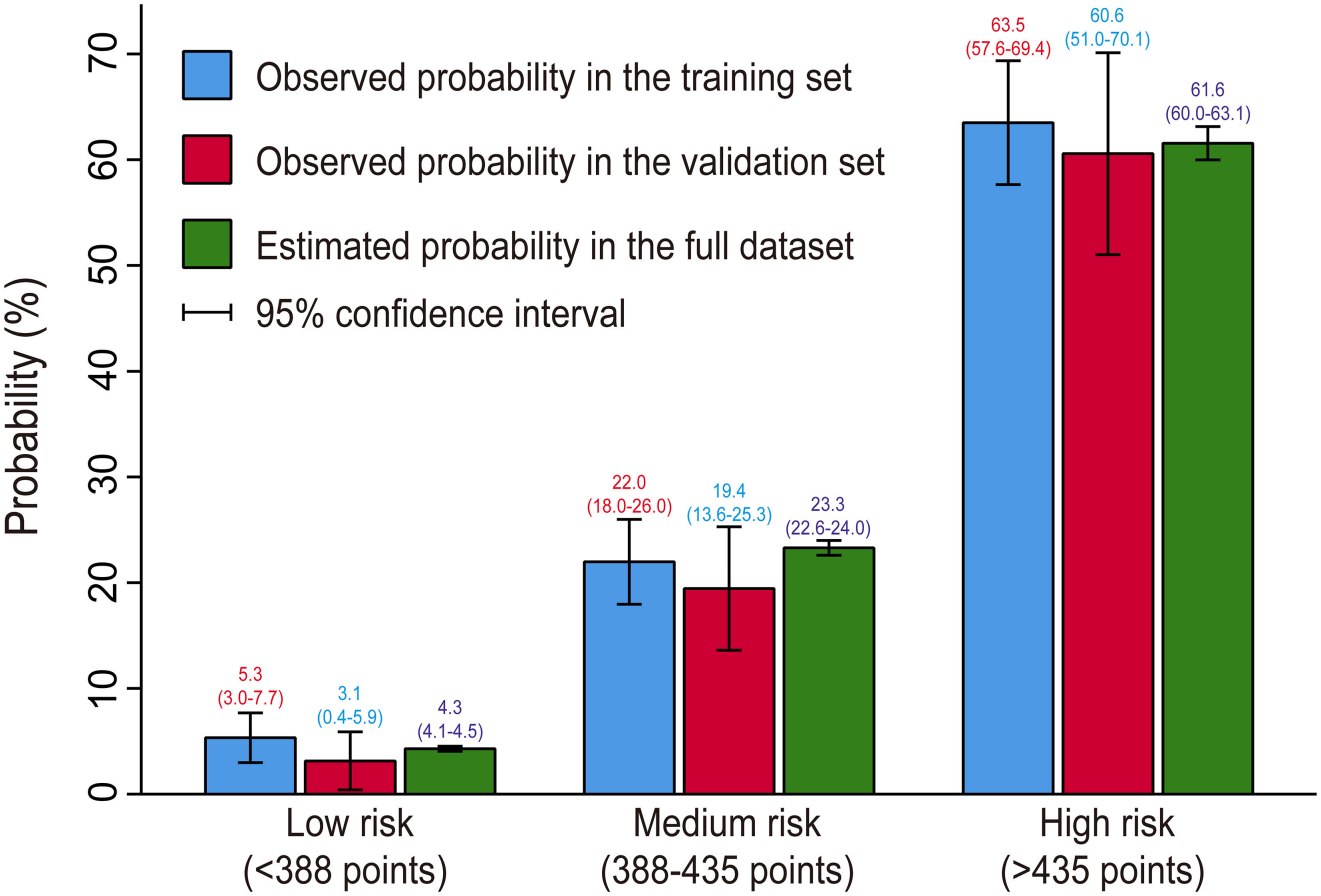
Bar chart showing the consistency between predicted and observed probabilities. No significant difference was observed between the predicted and actual probabilities in the same risk interval (*P* > 0.05) and it differed significantly between different risk intervals (*P* < 0.05), indicating good consistency and reasonable division.

We further performed a risk stratification on the basis of the full nomogram and clinical practice (Table 5). We selected predicted probabilities of 0.1 and 0.4 as the cutoff values and defined three risk groups as low, medium, and high risk groups, corresponding to the scores of <388 points, 388–435 points, and >435 points on the graphical nomogram. In this study, more than one-third of the patients were classified into the low risk group, about two-fifths into the medium risk group, and about a quarter into the high risk group. We compared the predicted probabilities and the observed probabilities in the training and validation sets between the three risk groups (Figure 5). No significant difference was observed between the predicted and actual probabilities in the same risk interval (*P* > 0.05) and it differed significantly between different risk intervals (*P* < 0.05), which indicated good consistency and reasonable division.

**Table 5:** Risk intervals of POH based on the nomogram.

**Risk intervals**	**Low risk**	**Medium risk**	**High risk**
	**(<388 points)**	**(388–435 points)**	**(>435 points)**
Estimated probability (%)	<10	10–40	>40
Observed probability, % (95% CI)	4.7 (2.8–6.5)	21.2 (17.9–24.5)	62.7 (57.7–67.6)
No. of patients (%)	515 (34.9)	594 (40.2)	367 (24.9)

*CI, confidence interval; POH, postoperative headache*.

### Outcomes

The overall mortality rate of the included patients was 8.7%, with a rate of 6.6% in patients without POH vs. 15.0% in those with POH [odds ratio (OR) = 2.510, 95% CI, 1.735–3.631; *P* < 0.001). We also observed significantly higher probabilities of readmission to ICU, reintubation and tracheotomy, and significantly longer postoperative ICU and hospital stay in patients with POH. Details of the comparison in patients with and without POH after AADS are presented in Table 6.

**Table 6:** Postoperative variables in patients with and without POH after AADS.

**Variables**	**All patients**	**Without POH**	**With POH**	***P* value**
	***n* = 1,476 (%)**	***n* = 1,096 (%)**	***n* = 380 (%)**	
Reintubation	216 (14.6)	122 (11.1)	94 (24.7)	<0.001
Tracheotomy	165 (11.2)	88 (8.0)	77 (20.3)	<0.001
Readmission to ICU	132 (8.9)	84 (7.7)	48 (12.6)	0.003
ICU stay (days)	7 (5, 11)	6 (5, 9)	9 (6, 15)	<0.001
Hospital stay (days)	21 (17, 27)	20 (16, 26)	24 (19, 31)	<0.001
Mortality	129 (8.7)	72 (6.6)	57 (15.0)	<0.001

*AADS, Stanford type A acute aortic dissection surgery; ICU, intensive care unit; POH, postoperative headache*.

## Discussion

POH has been reported to be an indicator of increased risk of mortality and averse outcomes (3, 5, 6), which was consistent with the present study. The overall incidence of POH after AADS was 25.7% and the mortality was 8.7%. However, compared with patients without POH, the mortality was significantly higher in patients who suffered from POH. Moreover, we observed significantly higher probability of other adverse outcomes such as tracheotomy and reintubation in these patients. The higher risk of adverse outcomes and mortality stressed the need of identifying independent risk factors and high-risk patients of POH after AADS.

Globally, various studies focused on identifying predictors for POH have been carried out in patients undergoing other surgeries (6, 8–10, 12), however, studies conducted in patients undergoing AADS are still lacking. To our knowledge, our work is the first report that describes the incidence, predictors, and outcomes of POH after AADS, and is the first attempt to construct and validate clinical prediction models in this area worldwide. In this study, we developed and validated a full nomogram model and a preoperative nomogram model using clinical data of 1,476 patients who underwent AADS in four tertiary care centers. The former was constructed based on five preoperative and three intraoperative predictors and the latter was based on seven preoperative predictors. Both nomogram models indicated good calibration, discrimination, and clinical usefulness. Three risk groups were finally divided to facilitate clinical application based on the full nomogram model and clinical practice.

Younger age and female sex have been reported to be associated with the development of POH in various surgeries (8, 9, 11, 14–16), which was consistent with our results. Droby and colleagues conducted a prospective study in patients who experienced post-lumbar puncture and found that compared to participants who did not develop a POH, patients that developed a POH were younger (*P* = 0.033) (17). Bitargil and colleagues conducted a respective study in patients undergoing endothermal ablation of the greater saphenous vein under spinal anesthesia and reported that female patients suffered from significantly more headaches than males (27 vs. 10%, *P* = 0.013) (9). Takenaka and colleagues conducted a large-scale study in patients who underwent primary lumbar spine surgery and reported that POH were more frequently observed in females than in males (OR = 2.70, *P* = 0.001) and advanced age was a significant protective factor for POH (OR = 0.70, *P* < 0.001) (14).

The sex- and age-related difference in POH are likely multifactorial. Recently, several potential mechanisms have been posited such as hormones influence, pain perception, and psychosocial factors (18). Fluctuating hormone levels during the menstrual cycle and hormone-related differences in cerebrovascular reactivity have long been blamed for the development of kinds of headaches (19–21). In terms of pain perception, it is believed that females and younger patients are more sensitive than males and older patients (22). In addition, compared to males and older patients, females and younger patients prefer to interpret, recall and report physical discomforts as symptoms such as pain, which may be due to the fact that it is more socially acceptable to express such emotions in those patients (16).

Chronic headache history was identified as an independent predictor for POH after AADS by multivariate analysis, which was in agreement with previous results in the literature (12, 15, 23, 24). Valentinis and colleagues conducted a prospective cohort study in patients who were operated on for intracranial tumors and reported that a longstanding headache history was the only significant independent intrapersonal predictor for POH (OR = 3.07, *P* = 0.01) (23). Ryzenman and colleagues conducted a large prospective cohort study in patients undergoing acoustic neuroma surgery to investigate the incidence and risk factors of POH and its effects on physical and psychosocial function (15). They found that preoperative headache was independently associated with the development of POH (OR = 1.4, *P* < 0.01) and patients who suffered from preoperative headache had more multiple occurrences of POH daily. Furthermore, Yabuki and colleagues reported that patients with preoperative headache had higher pain levels, higher neuropathic pain symptoms, and poorer quality of life (25). Hence, we believe that it may be an appropriate option to take prophylactic drugs to reduce the risk of POH for patients who had a chronic headache history (26).

Several other preoperative independent predictors for POH were also identified in our analysis, including cerebrovascular disease, smoking, WBC and platelet count. Headaches are common accompanying symptoms of cerebrovascular diseases and headaches attributed to ischemic strokes and transient ischemic attacks occur frequently. Oliveira and colleagues reported that the prevalence of headaches attributed to transient ischemic attacks and ischemic strokes were respectively 7.4–34% and 26–36% (27). A prospective study conducted by Matsota and colleagues indicated that smoking history was independently associated with the development of POH in patients undergoing elective surgery patients (OR = 1.74, *P* = 0.006) (8). Although the exact linkage between smoking and headache remains to unknown at present, it is undeniable that tobacco exposure is in some manner related to cluster headache (28). Given numerous negative health effects, decreased tobacco exposure and smoking cessation should be recommended in hopes of reducing disability and improving functionality (29).

The elevation of WBC count as a predictor for POH has been identified in previous study, which may relate to the acute phase systemic inflammatory response (24). Yazar and colleagues carried out a prospective study to investigate the role of inflammation and oxidative stress in the etiology of migraine (30). They found that the neutrophil, neutrophil/lymphocyte, monocyte/lymphocyte and platelet/lymphocyte ratios were higher in patients with migraine than patients without that (*P* < 0.05). The serum C-reactive protein, neutrophil, neutrophil/lymphocyte, monocyte/lymphocyte, and C-reactive protein /albumin ratios were higher during migraine attack periods (*P* < 0.05). Guo and colleagues conducted a retrospective study to explore prognostic factors for permanent neurological dysfunction after total aortic arch replacement with regional cerebral oxygen saturation monitoring. By multiple logistic regression analysis, they found that preoperative low platelet count was an independent predictor for postoperative neurological complications, which may be associated with platelet consumption coagulopathy (31). Siewert and colleagues conducted a genetic correlation analysis to explore risk factors for migraine headache using cross-trait linkage disequilibrium score regression and tested for potential causality between migraine and those phenotypes using Mendelian randomization (32). They found that migraine headache had genetic correlations with various traits including cardiovascular disease, smoking status, WBC count and platelet count.

Besides the preoperative predictors mentioned above, three intraoperative predictors for POH after AADS were also identified in this study, including RBC transfusion, CPB time and the use of DHCA. Although RBC transfusion can be lifesaving during cardiovascular surgery, massive transfusion has been confirmed to relate to various adverse events (33, 34). Arngrim and colleagues conducted a MRI spectroscopy and angiography study finding that experimental hypoxia was associated with headache attacks (35). Accordingly, we hypothesize that the reduced capacity of oxygen-carrying of the transfused RBCs which may lead to insufficient oxygen supply to the brain may be one of the responsible causes of POH. Furthermore, massive transfusion of RBC is often due to massive blood loss, which may also result in insufficient oxygen supply due to blood dilution and the reduction of active RBC and hemoglobin. Recently, a restrictive blood transfusion strategy has been especially recommended in clinical practice guidelines to prevent the development of various adverse events (36, 37).

It can be easily understood that CPB time is independently associated with the development of POH after AADS. On the one hand, longer duration of CPB often indicates longer duration of the whole surgery, longer forced position, and more intake of anesthetic agents, which have been reported to be significantly related to the development of POH (8, 38). On the other hand, the CPB process itself can result in brain injury and POH in many ways, including cerebral edema, embolism, hemodilution, and hypoxia (39–41). A systematic review conducted by Caldas and colleagues indicated that the development of neurological complications in patients who underwent cardiac surgery may be partially caused by the damage of cerebral autoregulation during CPB (40). Therefore, it may be effective to decrease the incidence of POH and other neurological complications through better brain protection strategy during CPB (41).

The use of DHCA has long been considered to be the standard neuroprotection strategy in patients undergoing AADS, however, this technique remains related to significant risk of brain damage and complications (42), which was again confirmed by this study. To reduce the risk of brain damage, continuous cerebral perfusion techniques have been proposed these years, including antegrade cerebral perfusion via the right subclavian artery only or with selective perfusion of both the carotid arteries and retrograde cerebral perfusion via the venous system. Using the UK National Adult Cardiac Surgical Audit, Benedetto and colleagues investigated the association between neuroprotective strategies and clinical outcomes in patients undergoing AADS (43). They found that compared to DHCA, the use of unilateral and bilateral antegrade cerebral perfusion was related to a decreased risk of death and cerebrovascular accident. Nonetheless, no consensus has been reached on which neuroprotective strategy should be preferred and there exists significant variation in clinical practice (44–47).

The nomograms may be helpful for risk evaluation, early prevention, surgeon-patient communication, and clinical decision-making. Taking appropriate strategies based on the nomograms may obtain more clinical net benefits. Besides preoperative precautions, preventive efforts during operations also make sense. Bezov and colleagues concluded that operator experience was a modifiable risk factors for POH (18). Benedetto and colleagues reported that high-volume surgeons, cardiac centers, and intraoperative factors were strong determinants of clinical outcomes after AADS (1).

There are several limitations in this study. First, the study was retrospectively designed and POH was diagnosed on the basis of medical records. Thus, we cannot assure that all the patients with POH were recorded in the database, which may result in an underestimation of the true morbidity. Second, some factors that may significantly relate to POH were not available in our analysis, such as operator experience. Nevertheless, the nomograms had reasonable performance in predictive capability, calibration, and clinical usefulness. Third, POH was the primary endpoint, but the evaluation of the types and severity of POH was not available because of the retrospective limitations. Prospective studies with more explicit classification of the types and severity of POH may make more sense in future work.

## Conclusions

To our knowledge, this is the first study describing the incidence, risk factors and outcomes of POH in patients undergoing AADS. POH was prevalent in our results, and the mortality and other adverse outcomes increased significantly in patients with POH. We constructed a full nomogram using five preoperative and three intraoperative predictors and a preoperative nomogram using seven preoperative predictors. Both nomograms demonstrated good calibration, discrimination, and clinical utility. We further defined three risk groups to facilitate clinical application. These findings may be helpful for risk evaluation, surgeon-patient communication, clinical decision-making, and early prevention.

## Data Availability Statement

The datasets presented in this article are not readily available because of patient confidentiality. Requests to access the datasets should be directed to the corresponding author.

## Ethics Statement

The studies involving human participants were reviewed and approved by the Ethics Committee of Tongji Medical College of Huazhong University of Science and Technology (IORG No. IORG0003571). Written informed consent for participation was not required for this study in accordance with the national legislation and the institutional requirements.

## Author Contributions

XD, XH, PY, and AZ: conception and design. XD, PY, HW, and SL: administrative support. XD, XH, HW, and AZ: provision of study materials or patients. DW, JW, FX, XL, SL, RL, YS, PY, and JL: collection and assembly of data. DW, SL, and XH: data analysis and interpretation. All authors: manuscript writing and final approval of manuscript.

## Funding

This work was supported by the National Natural Science Foundation of China (Grant Nos. 81800413, 81974048, and 81801586).

## Conflict of Interest

The authors declare that the research was conducted in the absence of any commercial or financial relationships that could be construed as a potential conflict of interest.

## Publisher's Note

All claims expressed in this article are solely those of the authors and do not necessarily represent those of their affiliated organizations, or those of the publisher, the editors and the reviewers. Any product that may be evaluated in this article, or claim that may be made by its manufacturer, is not guaranteed or endorsed by the publisher.

## Supplementary Material

Supplementary Figure 1 Screenshots of the interactive web-based dynamic nomogram created for the full predictive model of POH after AADS. Squares represent the point estimates for a given set of conditions, and the bars surrounding them reflect the 95% CIs. In the screenshots, we present the probabilities and corresponding 95% CIs of POH of 10 different patients **(A)**; when click the square, the information of the patient and corresponding risk are presented **(B)**; all the information of the patients can be acquired by clicking the “Numerical Summary” **(C)**; and the information of the model can be acquired by clicking the “Model Summary” **(D)**.

## Abbreviations

AADS: Stanford type A acute aortic dissection surgery
AUC: area under the receiver operating characteristic curve
CI: confidence interval
CPB: cardiopulmonary bypass
DHCA: deep hypothermic circulatory arrest
ICU: intensive care unit
OR: odds ratio
POH: postoperative headache
RBC: red blood cell
ROC: receiver operating characteristic
WBC: white blood cell.
