# Supplementary figures and images for: Incidence, Risk Factors and Outcomes of Postoperative Headache After Stanford Type a Acute Aortic Dissection Surgery
Source: Front Cardiovasc Med. 2021 Dec 23;8:781137. doi: 10.3389/fcvm.2021.781137 (PMC8733002; doi:10.3389/fcvm.2021.781137)

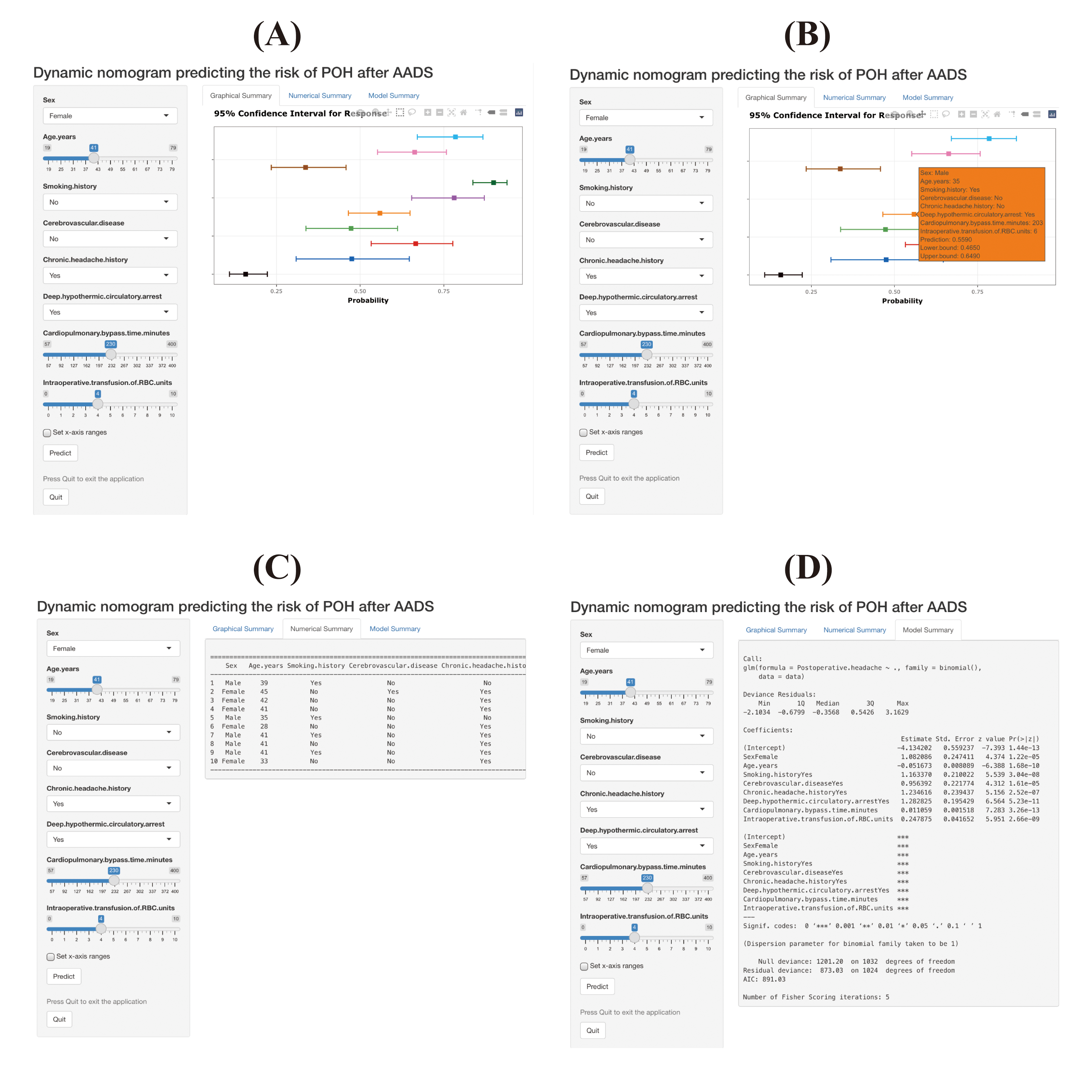

Supplement: Supplementary Figure 1 — Screenshots of the interactive web-based dynamic nomogram created for the full predictive model of POH after AADS. Squares represent the point estimates for a given set of conditions, and the bars surrounding them reflect the 95% CIs. In the screenshots, we present the probabilities and corresponding 95% CIs of POH of 10 different patients (A); when click the square, the information of the patient and corresponding risk are presented (B); all the information of the patients can be acquired by clicking the “Numerical Summary” (C); and the information of the model can be acquired by clicking the “Model Summary” (D). [file Image_1.TIF]
